# Heat Sepsis Precedes Heat Toxicity in the Pathophysiology of Heat Stroke—A New Paradigm on an Ancient Disease

**DOI:** 10.3390/antiox7110149

**Published:** 2018-10-25

**Authors:** Chin Leong Lim

**Affiliations:** Lee Kong Chian School of Medicine, Nanyang Technological University, Singapore 308232, Singapore; fabianlim@ntu.edu.sg; Tel.: +65-659-239-31

**Keywords:** heat stroke, endotoxemia, lipopolysaccharides, guts barrier, systemic inflammation, sepsis

## Abstract

Heat stroke (HS) is an ancient illness dating back more than 2000 years and continues to be a health threat and to cause fatality during physical exertion, especially in military personnel, fire-fighters, athletes, and outdoor laborers. The current paradigm in the pathophysiology and prevention of HS focuses predominantly on heat as the primary trigger and driver of HS, which has not changed significantly for centuries. However, pathological and clinical reports from HS victims and research evidence from animal and human studies support the notion that heat alone does not fully explain the pathophysiology of HS and that HS may also be triggered and driven by heat- and exercise-induced endotoxemia. Exposure to heat and exercise stresses independently promote the translocation of lipopolysaccharides (LPS) from gram-negative bacteria in the gut to blood in the circulatory system. Blood concentration of LPS can increase to a threshold that triggers the systemic inflammatory response, leading to the downstream ramifications of cellular and organ damage with sepsis as the end point i.e., heat sepsis. The dual pathway model (DPM) of HS proposed that HS is triggered by two independent pathways sequentially along the core temperature continuum of >40 °C. HS is triggered by heat sepsis at Tc < 42 °C and by the heat toxicity at Tc > 42 °C, where the direct effects of heat alone can cause cellular and organ damage. Therefore, heat sepsis precedes heat toxicity in the pathophysiology of HS.

## 1. Introduction

Heat stroke (HS) is the fatal form of heat injury that dates back more than 2000 years. In ancient times, HS was known to the Arabs as “Sariasis” after “Sirius”, which is the dog star that followed the sun in the summer [1,2]. Some scholars believe that the earliest documentation of HS is in the book of II Kings in the Old Testament where a Sunamite boy collapsed and died after complaining of a headache when working in the farm on a hot day [3,4]. We learn from military history that Roman troops were annihilated by HS in 24 BC during their expedition to Arabia [5,6]. In the 12th century, the English troops led by King Richard I also met the same fate with HS when fighting the Arabs for the holy land [4,7]. Closer to the present time, the Egyptian Army suffered more than 20,000 deaths allegedly due to HS in the Six-Day War against Israel in 1967 [7].

In spite of the long history, HS continues to threaten the health and safety of those who undertake physical work in modern times. Athletes, soldiers, fire-fighters, and outdoor laborers are among those who face a higher risk of HS because of the nature of their lifestyles and occupations [6,8,9,10]. During physical exertion, HS can occur even in cool weather conditions, which suggests that the intensity and duration of physical exertion are independent contributing factors in activating the mechanisms of HS and that a hot weather condition is not a pre-requisite for HS to take place [11,12].

Unlike advancements in other medical conditions where new discoveries from research led to more effective disease management outcomes, the paradigm on the pathophysiology and prevention of HS has remained relatively unchanged for centuries [3,6,13,14,15,16,17,18,19]. Both researchers and clinicians still subscribe to the concept that HS is triggered and driven primarily by heat when body temperature crosses a threshold, which is usually taken to be >40 °C [8,9,20,21,22,23]. Public health institutions and consensus statements from professional organizations such as the American College of Sports Medicine [24] and the National Athlete Trainer Association [25] also promote a heat-centered approach to prevent HS. These preventive measures are centered on avoiding a high body temperature during physical exertion by performing physical work within a permissible environmental temperature, including adequate fluid intake, wearing breathable clothing, and undergoing heat acclimatization [21,22,24,25]. This heat-centered approach for HS prevention continues to be promoted despite the continuing occurrence of HS and its related fatalities in sport and occupational environments.

HS continues to occur within the ambit of these heat-centered preventive measures including physical exertion in cool environmental conditions [10,12,26,27]. This author is not aware of any direct evidence showing that fluid intake can prevent HS. On the contrary, the experience of runners in road races and the running pace, and not fluid intake, were the key contributing factors to a high body temperature [28] and HS cases during endurance races [27,29]. Although heat acclimatization is effective in enhancing thermoregulation, the translation of improvements in thermoregulation to the prevention of HS remains debatable [30,31,32,33,34]. On the contrary, there are multiple reports of trained soldiers, outdoor laborers, and athletes who succumbed to HS [34,35,36,37,38,39], which suggests a dissociation between thermoregulation and heat tolerance. Two studies that administered continuous core temperature (Tc) measurement in well-acclimatized half-marathon runners showed that peak Tc in 30% to 40% of the runners were >40 °C (highest recorded was 41.7 °C) in the absence of heat injury or compromise in wellness [28,40]. The rectal temperature of runners measured at intervals and at the end of marathon races reached 41.1 to 41.9 °C without any symptoms of heat injury [41,42]. These data indicate that having a high Tc alone, up to about 42 °C, is physiologically tolerable and is not predictive of HS in trained and healthy individuals. This level of heat tolerance is higher than commonly perceived and is further supported by the relatively low HS incidence rate of about 0.02 [10]–5.6 [12] cases per 10,000 participants in endurance races. The contralateral implication of the low incidence rate for HS is that the majority of the runners could tolerate the same level of heat and physical stresses without succumbing to HS. Taken together, the current evidence suggests that Tc up to 42 °C can be tolerated by trained and healthy individuals and the reliability of heat stress exposure in predicting the risks of HS may not be as high as commonly suggested in the scientific literature [24,25].

There is a growing body of evidence in the last three decades that supports the notion that heat- and exercise-induced endotoxemia may play important roles in the pathophysiology of HS independently from the effects of heat stress [43]. For example, monkeys [44], rats [45], and dogs [46] were protected from lethal heat stress when endotoxemia was inhibited by pre-treatment with pharmaceutical and antibody agents, but animals in the control group died when endotoxemia was allowed to develop. HS victims also share similar clinical presentations with patients suffering from non-heat related sepsis [11,43,47]. Mild endotoxmia has also been reported in asymptomatic runners during endurance races [48,49,50]. The evidence presented suggests that endotoxemia may play important roles that are independent from heat in the pathophysiology of HS. Therefore, the aim of this review is to discuss the roles of endotoxemia and sepsis in the pathophysiology of HS and to suggest that heat-related sepsis precedes the thermolytic effects of heat i.e., heat toxicity, in the development of HS. The European Society of Intensive Care Medicine and the Society of Critical Care Medicine defined sepsis as “life-threatening organ dysfunction caused by a dysregulated host response to an infection” [51]. The systemic inflammatory response (SIR) is no longer a diagnostic criterion for sepsis but is one of the key drivers of the host response in a sepsis condition that leads to organ failures and other key clinical features of sepsis [51,52].

HS is classified into the “classical” and “exertional” forms, which are differentiated mainly by the source of heat that leads to a state of hyperthermia [22]. Hyperthermia in classical HS is due mainly to exogenous heat from the environment, which can occur in the absence of physical exertion, such as during heat waves [53]. The highest risk groups for classical heat stroke include the elderly-frail, infants, and toddlers. In contrast, the heat source for exertional HS is due mainly to endogenous heat, which is generated internally by the metabolic system during physical exertion. Although this review is conducted in the context of exertional HS, the evidence and information presented on the mechanisms of HS also apply to classical HS [53,54,55,56,57]. Therefore, this review will use the term “heat stroke” to refer to HS in general without differentiating between the forms of HS.

## 2. The Endotoxemia Model of Heat Stroke

The endotoxemia model of HS was first proposed by Moseley and Gisolfi [43] and further refined by Hales and Sakurada [58] and by Bouchama and Knochel [59] with some modifications. This model suggests that the pathophysiology of HS starts with a leaky gut, which results in the translocation of gram negative bacteria from the gut into the intravascular space [55,56,60,61]. The gut space is home to millions of bacteria from more than 500 species that could be characterized. Gram negative bacteria are harmful to the body because of an endotoxin unit located in the outer membrane of the bacteria [55,62]. Endotoxins are also known by its molecular structure as lipopolysaccharides (LPS), which comprise a core of oligosaccharides that is attached to a lipid A ligand and an O antigen [63,64].

The intestinal mucosa forms the gut barrier that separates the septic environment in the gut from the aseptic environment in the circulatory system [61]. The physical component (structure) of the gut mucosa is formed by the gut epithelium, which lines the luminal layer of the gut with a series of para-cellular tight junctions [43]. Under resting conditions, these tight junctions regulate intestinal absorption of fluid and nutrients from dietary intake and are impermeable to harmful substances, such as LPS, therefore, functioning as a barrier between the gut and the circulatory system [55]. Although a small amount LPS leaks across the gut barrier routinely, they are transported by portal circulation to the liver where they are removed from the body by Kupffer cells through phagocytosis [55,65,66,67].

The gut is very sensitive to both physical and psychological stressors, which explains the symptoms of spasm (known commonly as butterflies in the stomach), nausea, loss of appetite, and even diarrhea when exposed to varying degrees of stress [68]. Intense exercise is interpreted as stress signals by the gut and can cause damage to the gut lining and compromise the permeability of the gut barrier [55,61]. For example, up to 85% of runners have gastrointestinal symptoms, diarrhea, and intestinal bleeding during endurance races [69,70,71,72,73]. The shunting of blood away from the visceral organs, dehydration, and oxidative stress are possible mechanisms through which exercise and heat stresses compromise the structure and permeability of the gut [43,58,61,74,75,76,77]. The degree of increase in gut permeability is also positively associated with exercise intensity but is independent of heat stress [60,78]. The increase in permeability of the epithelial tight junctions allows bacteria and LPS to translocate across the gut barrier into the portal circulation where they are transported to the liver to be expelled from the body [55,79]. However, during a prolonged intense exercise, the rate of LPS influx into the liver can overwhelm LPS efflux, which causes LPS to overflow into the central circulation [58,61,65].

Within the central circulation, the next line of defense against LPS is dependent on monocytes from the innate immune system, high-density lipoproteins, and LPS-specific antibodies [62,63,64]. During prolonged intense exercise and when immune functions are compromised, the rate of LPS leakage can be greater than the removal of LPS by these anti-LPS mechanisms, which causes LPS to accumulate in the circulatory system [65,80]. LPS in the circulation can be harmful when they bind to cell surfaces through LPS binding proteins and when LPS presents itself as a pathogen associated molecular pattern (PAMP) [62,63,81]. There are different pathways through which LPS can be transported across the cell membrane, which are discussed in greater detail eslewhere [63,82,83]. In general, the PAMP signals from LPS activate toll-like receptor (TLR) 4, which is a pattern recognition receptor (PRR) on cell membranes. LPS is transported across the cell membrane by binding to TLR 4, which induces a cascade of downstream reactions that change the gene expression of the nucleus [63,81]. This chain of events activates the production of pro-inflammatory cytokines such as interleukin (IL)-6, IL-1β, and the tumor necrosis factor (TNF)-α. At a concentration threshold that is not determined presently, LPS can induce systemic inflammation and the septic response [63,83]. The downstream effects of SIR include disseminated intravascular coagulation, necrosis, cell death, and organ damage, which is consistent with the clinical presentations of sepsis and fatal HS [43,47,52,62,64]. To summarize, exercise and heat stress can cause endotoxemia by compromising the integrity of the gut barrier. In the blood, LPS penetrate cell membranes by binding to TLR 4, which induces pro-inflammatory cytokine production. Circulating LPS can accumulate to a threshold that activates SIR, which is the main driver for disseminated coagulation, necrosis, and organ failures, and which sets the condition for the development of sepsis. Therefore, the endotoxemia pathway of HS is mediated primarily by endotoxemia and SIR, which leads to sepsis as the end point.

## 3. Evidence Supporting the Endotoxemia Models of Heat Stroke

The fatality of HS limits the extent of human experimentation to empirically study the physiological mechanisms of this ancient illness. Research evidence on HS, including the endotoxemia model, is based primarily on pathological reports and clinical data of HS victims and animal experiments that involved lethal heat stress. Experiments on human subjects were done opportunistically on race participants and military personnel undertaking routine and self-selected activities. Laboratory experiments on human subjects conducted under sub-lethal heat load investigated thermoregulation responses and mechanisms and not heat tolerance and HS.

### 3.1. Pathological Reports and Clinical Data

One of the earliest evidence to suggest the possible roles of SIR and sepsis in HS came from clinical observation of Israeli soldiers who suffered HS between 1955 and 1965 [11] and from American soldiers who succumbed to HS in World War II [6,15]. The clinical findings in these HS victims included increased blood concentration of liver enzymes that are associated with tissue damage (e.g., serum glutamic oxalacetic transaminase, lactate dehydrogenase, and creatine phosphokinase), disseminated intravascular coagulation and necrosis that led to wide spread cellular and multi-organ damage, which are consistent with the ramifications of SIR and sepsis. These clinical features of HS victims were also reported in nine soldiers and one race participant who died of HS in Singapore [47] and in civilians who were treated and died of HS at hospital emergency departments [54,57]. Muslim pilgrims who were admitted to the Emergency Department in Mekka also had elevated concentrations of circulating LPS, IL-6, TNF-α, and IL-β [84,85]. These clinical findings on HS victims share many common features with sepsis patients from non-heat related infection and support the notion that immune disturbances involving systemic inflammation and sepsis play critical roles in driving the pathophysiology of HS.

### 3.2. Animal Studies

Research evidence supporting the endotoxemia model of HS was also derived from animal studies. For example, reducing the stool content and the gut flora in dogs by a mix of antibiotics and dietary interventions protected 70% of the animals during lethal heat stress exposure, which was compared with only a 20% survival rate in the control group [46]. In sedated rats, gut permeability increased linearly as heat exposure increased Tc from 37 to 42.5 °C, which resulted in severe damage to the gut wall [56]. Another series of studies treated sedated monkeys with LPS-antibodies, antibiotics, and corticosteroids before they were heated in an incubator until Tc was 43.5 °C [44,86,87]. These treatments inhibited endotoxemia and protected all the animals from lethal heat stress. In contrast, plasma LPS concentration of animals in the control group increased markedly, which resulted in 70% to 80% mortality. In another group of monkeys, both the animals treated with anti-LPS antibodies and those in the control group died when Tc was increased to 43.8 °C [87]. However, animals in the treatment group survived 5.3-fold longer under lethal heat stress than animals in the control group. The concentration of plasma LPS also increased significantly in the control group but was unchanged from the resting concentration in the treatment group.

The effects of physical fitness on heat tolerance was investigated in sedentary and aerobically-trained Merino sheep [88]. Tc was significantly lower in aerobically-trained animals than in sedentary animals during lethal heat exposure (Tc 42 °C). Aerobic fitness also resulted in higher cardiac output, which mitigated the reduction in blood flow to the gut and major organs during lethal heat exposure. However, there was no difference in the Tc response under the same heat load when the sedentary sheep were treated with indomethacin prior to heat exposure. These results imply that a higher level of aerobic fitness can contribute to heat tolerance and thermoregulation through improvements in cardiovascular functions. The modulation of the Tc response in the sedentary sheep by indomethacin suggests that there are common pathways regulating body temperature during heat exposure and the pyrogenic pathway that induces fever. Since indomethacin blocks the prostaglandin pathway in endotoxin-induced fever, these results also imply the potential roles of endotoxemia in the mechanisms of HS [88].

The author of this review subjected sedated rats to intramuscular injection of turpentine to induce aseptic inflammation, intraperitoneal injection of dexamethasone to block endotoxemia, and a combination of the turpentine and dexamethasone injections [45]. Animals in the control group received an equal volume of intraperitoneal saline injection. Two hours after administering the respective treatments, the animals were heated with an infrared lamp until Tc was 42 °C for 15 min. A single dose of dexamethasone inhibited the increase in plasma LPS concentration and protected all the animals in the group from lethal heat stress. However, plasma LPS concentration in the control and the turpentine treatment groups increased significantly during lethal heat stress, which resulted in 30% to 40% mortality. Plasma LPS concentration in the group that received both dexamethasone and turpentine treatments were unchanged from resting concentration, but this group had the highest mortality of 67%. The plasma concentrations of alanine aminotransferase transaminase (ALT), aspartate transaminase (AST), IL-6, IL-1β and TNF-α increased significantly in all the groups, except the dexamethasone group, which were unchanged from pre-heating concentrations. The combination of turpentine and dexamethasone treatments also resulted in the highest increase in the plasma concentrations of ALT and AST, which are biomarkers for tissue damage. Taken together, evidence from these animal studies suggests that endotoxemia may function as an independent switch that activates the pathophysiology of HS. This role of endotoxemia in causing HS takes place in the presence of lethal heat stress but is functionally independent from the effects of heat toxicity. However, under conditions of pre-existing inflammation (turpentine) or extreme heat load (>43.5 °C in monkeys), the toxic effects of heat alone can trigger the mechanisms of HS in the absence of endotoxemia. Contrary to the long-held consensus that HS is triggered mainly by heat stress, the evidence presented supports the notion that, besides heat, HS may also be triggered independently by endotoxemia and mediated by SIR to result in sepsis.

### 3.3. Human Studies

The possible roles of gut-related endotoxemia in the pathophysiology of HS have also been studied in human volunteers. Experiments that used ingested sugar probes of varying sizes (lactulose, rhamnose, and sucrose) to measure gut permeability showed a positive relationship between the increase in gut permeability and running intensity at 40%, 60%, and 80% of peak volume of oxygen uptake [78]. The first report on exercise-induced endotoxemia was from Brock-Utne and colleagues (1988) who found that increased concentration of plasma LPS was present in 83% of 89 runners in the 89.4 km Comrade Run [89]. About 40% of runners with higher plasma LPS concentration had gastro-intestinal symptoms of nausea, vomiting, and diarrhea. In the same year, the same group of investigators reported a 3.6-fold increase in plasma LPS concentration, from 0.081 ng/mL before the race to 0.293 ng/mL after the race, in 18 triathletes [90]. The resting plasma LPS concentration in these triathletes was positively associated with their training intensities, which supports the notion of a dose response relationship between exercise intensity and LPS leakage into the central circulation. With the availability of more sensitive bioassay kits for LPS, lower concentrations of 0.05–15 pg/mL increases in plasma LPS concentration were reported in a half-marathon [91], marathon [49], and triathlon [50]. In a laboratory experiment [92], trained and untrained participants performed treadmill walking in protective suits in a heat chamber (40 °C, 30% relative humidity) to achieve a Tc of 39.5 °C. Blood samples were taken before the exercise and at 0.5 °C intervals from Tc 38 to 39.5 °C during the experiment. Increased concentrations of plasma LPS were detected at Tc 38 °C and plasma LPS concentration was two-fold higher than resting concentration when Tc reached 39 °C. The peak plasma LPS concentration was 16.4 pg/mL in the trained group and 34 pg/mL in the untrained group, which supports the benefits of physical fitness in mitigating exercise-induced endotoxemia. Collectively, the evidence presented showed that exercise and heat stresses can result in mild endotoxemia, which was associated with increased plasma concentrations of IL-6, IL-1β, TNF-α, and nuclear factor kappa B cells (NFкB) and decreased plasma concentrations of LPS antibodies known as immunoglobin (Ig) G and IgM. The increase in permeability of the gut was also positively associated with exercise intensity and the state of training, which suggests the existence of cross-talk mechanisms between the gut and the exercise stress signals.

In spite of the growing body of research evidence in the last three decades supporting the roles of endotoxemia, systemic inflammation, and sepsis as an independent pathway in the pathophysiology of HS, the consensus among scholars and clinicians continues to focus on heat as the primary trigger and driver of HS [21,24,25,93]. One proponent of the endotoxemia model postulated that HS is triggered by heat but is driven by endotoxemia [43]. Once the mechanism of HS is triggered, the progress in the pathophysiology of HS is driven independently by the degree of systemic inflammation and severity of the sepsis condition and not driven by the degree of heat stress. With the benefits of new research evidence and the body of knowledge from Exercise Immunology research relating to exercise-induced immune suppression, Lim and Mackinnon [65] proposed the Dual Pathway Model (DPM) of HS to achieve better alignment between research, field, and clinical evidence. It is not within the scope of this review to discuss Exercise Immunology research, but readers are referred to several excellent reviews on this topic for further reading [80,94,95].

## 4. The Dual Pathway Model of Heat Stroke

The DPM suggests that HS is triggered by two independent pathways that are activated sequentially [65]. The first pathway is due to endotoxemia, systemic inflammation and the sepsis response, and is labelled as the “heat sepsis” pathway. The mechanisms and research evidence supporting the roles of endotoxemia, systemic inflammation, and heat sepsis in the pathophysiology of HS have been discussed above. Although heat sepsis triggers HS under a state of hyperthermia, the mechanisms of heat sepsis is functionally independent from the thermolytic effects of heat [40,65,86]. The contribution of heat in this pathway may be limited to the induction of gut permeability changes and in promoting LPS translocation into the circulatory space [60]. The second pathway in the DPM is due to the thermolytic effects of heat, which is also known as the heat toxicity pathway, where high temperature alone can cause disintegration and damage to cellular structures and organs in the body [31,96,97,98,99,100,101,102]. The DPM proposed that HS is triggered sequentially along the Tc continuum > 40 °C with the heat sepsis pathway preceding the heat toxicity pathway.

The transition between the heat sepsis and toxicity pathways is postulated to be around Tc 42 °C. This postulation is based on evidence from endurance race participants who, barring any illness, tolerated a Tc of up to 42 °C without any health or physiological consequences [40,42]. The 42 °C threshold is also consistent with the critical thermal maximum (CTM) for humans, which was estimated to be between Tc of 41.6 °C [103] and 42 °C [4]. The CTM is the lowest high deep body temperature that is lethal to an animal [104] or the threshold above which fatality may occur. The CTM threshold was studied in sedated cancer patients who were heated to a Tc of 41.8 °C for an hour [105] and to 42 °C for up to 8 h [106,107] as a treatment modality. None of the patients in these studies suffered from any adverse effects of the heat exposure. Since a Tc of up to 42 °C is well tolerated in these human studies, the occurrence of HS within this temperature range would be due to the heat sepsis and not the heat toxicity pathway. 

HS can also be caused by the heat toxicity pathway if Tc progresses to >42 °C where the thermolytic effects of heat alone can trigger the pathophysiology of HS. Fibrinolysis, hemolysis, and the inability of platelets to aggregate have been reported at this level of heat stress [57,108,109]. Extreme heat stress can also liquefy the cell membrane and damage the cellular structure and organelles [96,100,110]. The combination of these toxic effects of heat can lead to multiple organ failure, which is commonly observed in heat stroke victims [11,15,47]. These heat-induced damages to the cells and organs in the body are consistent with the effects of heat toxicity in the second pathway of the DPM.

The suggestion of 42 °C as the Tc cross-over point between the two DPM pathways should be taken conceptually and not as an absolute and fixed cross-over point in the DPM. This Tc threshold is likely to vary to some extent due to differences in individual physiology and the state of health and fitness. An overlap between the two DPM pathways is highly possible. However, this overlap is likely to be only in the direction of the sepsis pathway crossing into the heat toxicity pathway at Tc > 42 °C and not the other way around. This direction of overlapping pathways is because LPS and SIR can continue to be active in both the lower and upper ranges of the Tc continuum > 40 °C, but heat toxicity can only operate in the upper range of >42 °C to have a sufficient heat load to cause cellular and organ damage. In summary, the DPM suggests that, besides heat, HS can also be caused by the effects of endotoxemia, systemic inflammation, and sepsis i.e., the heat sepsis pathway. Heat sepsis precedes heat toxicity in causing HS and the transition between these two pathways is postulated to be around Tc 42 °C, which is the threshold for activating the heat toxicity pathway.

**Figure 1:**
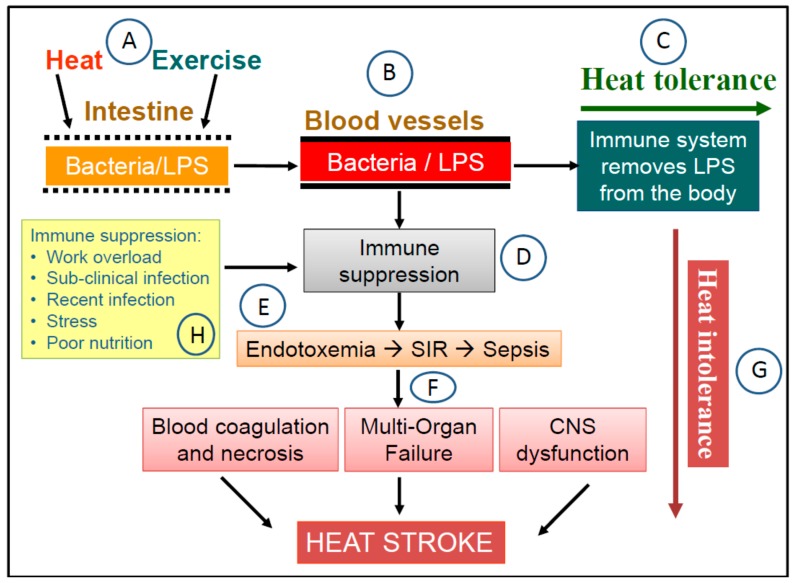
The heat sepsis pathway in the dual pathway model (DPM) of heat stroke (HS). (**A**) Exercise and heat stresses cause the permeability of the gut epithelium to increase, which leads to the leakage of gram-negative bacteria and lipopolysaccharides (LPS) from the gut into the circulatory system (**B**). In a healthy state, when immune functions are not compromised, LPS in the circulation is removed from the body by monocytes, high density lipoproteins, and LPS-specific antibodies. Under these circumstance, the physical task is completed without heat-related health consequences i.e., heat tolerance (**C**). However, when undertaking the same physical task in a state of immune suppression (**D**) can compromise LPS clearance, which leads to the accumulation of LPS in the blood i.e., endotoxemia (**E**). The concentration of LPS in the blood reaches a threshold that triggers the systemic inflammatory response (SIR), which can lead eventually to sepsis. The resulting clinical presentation include massive blood coagulation, necrosis, cellular damage, multi-organ failure, and central nervous system disturbances (**F**), which are seen in HS victims i.e., a state of heat intolerance (**G**). In this pathway, the state of the immune system can function as a switch between heat tolerance and intolerance i.e., transient heat tolerance. Examples of circumstances that can cause immune suppression are also suggested in the diagram (**H**).

There is good agreement in Exercise Immunology research that prolonged intense training (or physical exertion) without a sufficient opportunity for recovery can result in short term to chronic immune suppression and chronic mild inflammation [95,111,112]. Since LPS influx and removal are key determinants of the development of endotoxemia, this evidence implies that heat tolerance or intolerance in the sepsis pathway may be transient, depending on the state of immune functions and gut health at the point of physical exertion. The same individual who can tolerate the work and heat load on one occasion may suffer HS due to heat sepsis on another occasion under the same conditions when immune functions and/or gut health are compromised. This concept of transient heat tolerance in the sepsis pathway is illustrated in Figure 1 and may explain the occurrence of HS in people who are well-trained and when performing activities that were well-tolerated before [34,38]. Besides exercise-induced immune suppression, immune functions can also be compromised under other circumstances such as the existence of a sub-clinical infection, recent illness, and prolonged periods of psychological stress, poor nutrition, and sleep deprivation [113,114,115,116]. Therefore, the protection of gut, the immune system, and the overall state of health is a key foundation in protecting again HS due to heat sepsis.

The concepts of the DPM should not be misinterpreted to suggest a lesser emphasis on heat stress in the prevention of HS. HS cases occur at Tc > 40 °C and the risk of having HS increases at and above this level of heat stress, which does not change with the DPM [117]. The novelty of the DPM is in recognizing the non-heat related causes of HS in the Tc continuum > 40 °C. The DPM promotes giving equal emphasis to the level of heat stress and to protecting against the development of endotoxemia in the prevention of HS. Since the heat sepsis pathway originates from the leakage of gut-related LPS, the prevention of HS should include protecting the immune system, the integrity of the gut barrier, and the overall state of health, especially during periods of intense physical training and when immune functions and gut health can be compromised.

### Potential Mitigation of Heat Sepsis

The risk of having HS through the heat sepsis pathway may be mitigated by limiting the increase in LPS concentration both in the liver and in the central circulation and by inhibiting SIR [45,87] and protecting immune functions during periods of intense physical exertion [45,86,118]. Antibiotics are potentially effective in limiting LPS translocation in the gut due to its antimicrobial properties and a single dose of Kanamycin was shown to protect monkeys from lethal heat stress of up to Tc 42 °C [87,119]. However, the use of antibiotics as a prophylaxis against heat sepsis is not feasible because the antimicrobial actions also destroy “good” bacteria that contributes to a healthy balance of bacteria profile in the gut flora [120]. Frequent use of antibiotics also leads to antibiotics resistance, which limits the potential use of antibiotics for the treatment of other infections.

Non-steroidal anti-inflammatory drugs (NSAIDS) are used frequently by athletes to reduce pain and joint inflammation following intense training [121,122,123,124]. The same anti-inflammatory properties in NSAIDS may potentially be extended to block the development of SIR during heat stroke. However, the use of NSAIDS can lead to gastrointestinal injury [50,125] and contribute to the loss of gut permeability [126]. NSAIDS also induce splanchnic hypoperfusion, which further compromise the integrity of the intestinal barrier [120]. For example, treating mice with 5 mg/kg of indomethacin before heat exposure resulted in 45% mortality, damage to the gut barrier, and hemorrhage 24 h following lethal heat exposure (Tc 42.4 °C) [127]. In humans, ingesting 600 mg (one day before race) and 1200 mg (on race day) of ibuprofen before a 160-km ultra-marathon resulted in higher plasma concentration of LPS and pro-inflammatory cytokines and macrophage inflammatory protein 1β at the end of the race [128]. The evidence presented suggests that NSAIDS have limited prophylactic value in mitigating the risks of heat sepsis and may cause further damage to the gut barrier.

Besides pharmaceutical agents, dietary supplementation with probiotics and bovine colostrum may offer some protection against the development of heat sepsis during intense exercise and heat exposure. Probiotics are the “good” bacteria that offer health-promoting benefits and they are dominated mainly by the lactobacilli and bifido strains of bacteria [129]. The main benefits of probiotics are due to its anti-inflammatory effects in the gut, which is the underlying cause of gut-related diseases e.g., colitis and inflammatory bowel disease [130]. Probiotics also stimulates the secretion of mucin in the epithelial cells, which inhibits the adherence of pathogenic microbes to the gut wall and contributes to gut-mucosal immunity [131,132]. The benefits of probiotics are well established in in vitro and whole-animal studies and in patients with bowel-related diseases. The benefits of probiotics supplementation in protecting the gut barrier and in limiting exercise-induced endotoxemia are less clear in human studies. For example, four weeks of probiotics supplementation (45 billion/day) had no effects on gut permeability and plasma LPS concentration during intense exercise in the heat [79]. Although probiotics are potentially beneficial in protecting the integrity of the gut barrier and in limiting LPS translocation, the current evidence does not support the translation of these benefits to human applications during exercise and heat exposure.

Bovine colostrum is the first volume of milk produced by cows after parturition. The health benefits of colostrum, in general, are found in the abundance of immune cells and growth factors and in its antimicrobial properties, which are pertinent for the protection and growth of neonates [133]. Bovine colostrum has higher concentration of these biological and health-promoting ingredients than human colostrum and is used by athletes to protect immune health and for enhancing sports performance e.g., muscle cell regeneration and growth [133]. Bovine colostrum supplementation was effective in limiting the increase in gut permeability induced by NSAIDS [134,135,136] and intense exercise [137] and in protecting against gastrointestinal damaged due to the effects of transforming growth factors-β [134]. Ingesting 26 mg/day to 60 mg/day of bovine colostrum for eight to 12 weeks was independently beneficial in reducing the incidence of the upper respiratory tract infection and in increasing salivary immunoglobin-A concentration [138,139]. These results suggest that bovine colostrum may offer protection against heat sepsis by contributing to LPS removal during exercise and heat exposure. However, there are contradicting reports on the effects of bovine colostrum supplementation on gut permeability during exercise. For example, one study showed increased gut permeability during intense exercise following eight weeks of supplementation [140] and another study showed no effects of bovine colostrum on gut permeability during exercise in the heat after seven days of supplementation [141]. These results contradicted the finding that two weeks of supplementation with bovine colostrum modulated the increase in gut permeability during intense exercise [137]. These differences in results may be due to variations in the dosage and period of supplementation and also to the different experimental conditions in these studies. Nevertheless, the rich nutritional properties of bovine colostrum are potentially beneficial to gut health and immune functions, which deserve further investigation. A well-controlled study on the dose-response relationship and on the effects of duration of bovine colostrum supplementation on gut and immune functions during exercise and heat exposure would provide important information on the potential use of bovine colostrum as a countermeasure against heat sepsis. If proven to be useful, bovine colostrum can be practically administered at the individual and group levels to promote health and to mitigate the risks of heat sepsis during periods of intense physical exertion.

Beyond the use of pharmacological and nutritional products, the mechanisms of heat sepsis may also be mitigated through behavioral measures that minimize the opportunity for immune suppression. Twelve years of medical records in a military hospital in Thailand showed that 96% of soldiers who had HS experienced a bout of mild fever, 16% had an upper respiratory tract infection, and 3.4% had diarrhea before the HS event [142]. Recurrent HS was also associated with a bout of fever and gastroenteritis [143]. These studies demonstrated the close association between a sub-optimal immune function and the occurrence of HS, which support a more holistic approach to lower the risks of HS by maintaining a good state of health especially during periods of intense physical exertion.

In a routine lifestyle, having adequate and regulated rest and sleep and consuming a balanced diet with sufficient macro-nutrients and micro-nutrients are the foundation of good health. People who undertake strenuous activities in sports and occupational settings should also monitor for early symptoms of illness and take the appropriate measures to adjust the workload until these symptoms are adequately resolved [115]. Athletes undertaking a prolonged period of intense training should have dedicated plans to prevent overreaching and overtraining. These plans would include, among other measures, a periodization plan that provides adequate opportunities for recovery, which is interfaced with intense training and competition [144,145]. The risks of heat sepsis can be reduced by protecting immune functions during periods of an intense physical workload.

## 5. Future Research

The DPM of HS was first published in 2006 [65] as an extension of the endotoxemia model of HS, which was first published in 1993 [43]. The body of evidence supporting the concepts of the DPM has grown since it was first introduced (discussed above). More recently, an extensive review by Armstrong and colleagues [83] alluded to the potential roles of gut bacteria in the mechanisms of HS including the potential roles of the gut microbiomes as a whole and not just gram negative bacteria in causing HS. However, there are still gaps in the understanding of the DPM and mechanism of HS, in general, that deserve further research.

Although there is strong evidence on changes in gut permeability and damage to the gut barrier during exercise and heat exposure, the exact mechanisms causing these changes in the gut are not well understood. The potential roles of gut microbiomes in promoting and inhibiting the mechanisms of HS also deserves more attention. A better understanding of the gut barrier response and the roles of gut bacteria in the development of HS can potentially lead to more options for mitigating the risks of heat sepsis through improvements in gut health. More insights are also needed on the mechanism for transporting LPS across cell membranes and the downstream effects on cytokine secretion. This understanding of LPS-cell interaction may be useful for the development of prophylaxis to block the LPS insult during prolonged exercise and heat exposure. Another area that require more investigation is the Tc cross-over point and overlaps between heat sepsis and toxicity, which remains a postulation based on other indirect evidence. Better understanding of the interaction of the heat sepsis and toxicity pathways along the Tc continuum and the response of this interaction under different health and fitness statuses can be useful for the development of more effective HS mitigation strategies. Another important area of research is in the plasma LPS concentration threshold for triggering the SIR. Although the lethal concentration threshold for endotoxemia is 1 ng/mL [146], the LPS concentration threshold for triggering the systemic inflammatory response is unknown presently. Understanding this threshold and the factors that influence the shifts and stability of the threshold can provide a good benchmark/target for blocking the progression of endotoxemia to systemic inflammation and the downstream consequence of sepsis.

A new area of consideration for the DPM is the potential link of heat sepsis and toxicity with the endocrine system, especially in protecting central nervous functions during lethal heat exposure [147]. For example, compared with healthy individuals, HS patients had higher plasma concentration of beta-endorphine, which is involved in thermoregulation, during a heat tolerance test [148]. In animals, dexamethasone was effective in protecting rats [45] and monkeys [149] from endotoxemia and lethal heat stress. Dexamethasone is a glucocorticoid hormone, which is used for the treatment of cerebral ischemia and spinal cord injury. Treating rats with dexamethasone before heat exposure and immediately after the onset of HS resulted in lower serum concentration of IL-β and mitigated HS-induced arterial hypotension, cerebral ischemia, neuronal damage, and prolonged the period of survival after the onset of HS [150]. However, treatment of baboons with dexamethasone before heat exposure and after the onset of HS aggravated the degree of tissue damage, multi-organ failure, and death due to HS [151].

Besides glucocorticoids, melatonin may also enhance protection against inflammation and oxidative stress insults during heat stroke [147]. In rodents, treatment with melatonin immediately after onset of HS was effective in reducing the magnitude of inflammation, edema, hemorrhage, and oxidative and organ damages in a dose-response manner in the lungs, brain, and other major organs in the body [152,153]. These results suggest that the endocrine system may play important roles in protecting the major organs from the effects of heat sepsis and toxicity after the onset of HS. However, most of the evidence on the protective roles of the endocrine system in HS were done on animals that investigated the protective effects of these hormones after the onset of HS and not on their roles in triggering and driving the mechanism of HS. More research is needed to shed more light on the roles of the endocrine system in the mechanisms of HS.

## 6. Conclusions

HS has been a health threat during physical exertion for more than 2000 years, but the approach in HS prevention has not changed significantly for centuries. The continuing occurrence of HS cases and fatalities in modern times suggests that there are non-heat related factors causing HS that have not been adequately addressed. The DPM is an extension of the endotoxemia model of HS that aims to provide greater clarity on the independent roles of heat sepsis and toxicity in pathophysiology of HS. The concept of “heat sepsis” was also introduced to better reflect the endotoxemia-related mechanisms in the pathophysiology of HS. The inclusion of evidence from exercise immunology research adds a novel dimension of immune suppression to the endotoxemia model. The inclusion of immune suppression in the HS paradigm more accurately reflects the compromise in host-defense mechanisms against exercise-induced endotoxemia resulting from prolonged exposure to intense physical work. Hopefully, the novel concepts introduced by the DPM of HS can stimulate more research and academic debates that will lead to better understanding of HS mechanisms and a reduction in the incidence and fatality of HS cases globally.
